# Formulation of Novel Liqueurs from Juice Industry Waste: Consumer Acceptance, Phenolic Profile and Preliminary Monitoring of Antioxidant Activity and Colour Changes During Storage

**DOI:** 10.17113/ftb.59.03.21.6759

**Published:** 2021-09

**Authors:** Marija Petrović, Sonja Veljović, Nikola Tomić, Snežana Zlatanović, Tomislav Tosti, Predrag Vukosavljević, Stanislava Gorjanović

**Affiliations:** 1 Institute of General and Physical Chemistry, Studentski trg 12, 11158 Belgrade, Serbia; 2 Faculty of Agriculture, University of Belgrade, Nemanjina 6, 11080 Belgrade, Serbia; 3 Faculty of Chemistry, University of Belgrade, Studentski trg 12-16, 11080 Belgrade, Serbia

**Keywords:** pomace liqueur, antioxidant activity, phenolic profile, sensory analysis, circular economy in juice production

## Abstract

**Research background:**

Apple juice is one of the most popular and liked beverages worldwide. Due to the increased health consciousness among consumers, beetroot and chokeberry juices have also rising consumption trends. Despite representing a considerable percentage of the processed fruit and rich source of bioactive compounds, fruit pomace, remaining after juice production, has still been underutilised. Here, the possibility of using apple, beetroot and chokeberry pomace in liqueur formulations is investigated.

**Experimental approach:**

Apple and chokeberry liqueurs were produced from apple and chokeberry pomace extracts, respectively. Apple/chokeberry and apple/beetroot liqueurs were obtained by combining apple pomace with chokeberry and beetroot pomace extracts in ratios 50:50 and 70:30, respectively. The sensory quality and acceptability of freshly prepared liqueurs were evaluated by experts and consumers. Sugars and phenolics were identified and quantified by high-performance anion-exchange chromatography with pulsed-amperometric detection (HPAEC-PAD) and high-performance liquid chromatography–diode array detection–tandem mass spectrometry (HPLC–DAD–MS/MS), respectively. Storability was preliminarily evaluated based on monitoring of total phenolic concentration, antioxidant activity and colour each month during 6 months of storage at 4 and 22 °C.

**Results and conclusions:**

The expert and the consumer testing indicated that apple and chokeberry pomace could be used as raw materials without any flavour corrections while apple/beetroot pomace liqueur would require modification. High total phenolic content and antioxidant activity were found in all freshly prepared liqueurs, with chokeberry liqueur being by far superior. Among identified phenolics, ellagic acid and phlorizin were quantified as the most prominent, except in chokeberry liqueur, where phlorizin was not quantified. Despite the decrease in total phenolic concentration and antioxidant activity after 6 months, liqueurs still represented a rich source of phytochemicals. The highest phenolic compound retention and antioxidant activity maintenance were observed in chokeberry liqueur. Also, the appealing colour was retained despite the changes detected in chromatic characteristics.

**Novelty and scientific contribution:**

The possibility of apple, beetroot and chokeberry pomace restoration into the food chain by the production of liqueurs has been demonstrated for the first time. Functional and sensorial properties of newly developed liqueurs indicated that the selected pomace represents the promising raw material for liqueur production. The applied approach represents a contribution to the circular economy in juice production.

## INTRODUCTION

One of the most promising waste materials from the food industry is pomace, a by-product in juice production, which mainly contains skins, pulp, seeds and stalks of the fruit. Phenolic compounds are mainly found in fruit skin as natural plant protection from environmental factors, so pomace is a valuable source of polyphenols, especially if taking into account that most of the antioxidants tend to stay in the pomace rather than transfer into juice (*1*, *2*). According to the data for 2016, the EU-28 countries produced roughly 2.1 billion litres of apple juice (*3*). Apple and beetroot are commonly used, while chokeberry use in juice production is constantly increasing.

Apple pomace makes up to 25–35% of the processed fruit (*4*). Phenolic compounds (catechins, procyanidins, phloridzin, phloretin glycosides, caffeic and chlorogenic acid, quercetin and cyanidin glycosides) and dietary fibre (soluble pectins, β-glucans, galactomannan gums, nondigestible oligosaccharides including inulin and insoluble lignin, cellulose and hemicelluloses) of apple pomace exhibit antioxidative, cardioprotective, antidiabetic and antilipemic effects and improve the function of the gastrointestinal tract. Only 3-10% of the overall antioxidant activity of an apple remains in the apple juice. However, apple pomace is still used only as animal feed in Serbia (*5*, *6*). Despite numerous health benefits and high potential for utilisation as a substrate, source of bioactive compounds or ingredients of various food products, this abundant, available and renewable natural resource is still underutilised.

Beetroot is one of the ten most powerful vegetables in terms of antioxidant capacity. Beetroot juice production yields about 15–30% of beetroot pomace. Beetroot pomace obtained from different cultivars from Serbia was reported to contain ferulic, vanillic, *p*-hydroxybenzoic, caffeic and protocatehuic acids and betalains (betanin, isobetanin and vulgaxanthin I) (*7*). These compounds possess many properties beneficial to health, including free radical scavenging ability. Total phenolic content decreases in the order: peel (50%), crown (37%) and flesh (13%), which is evidence of a considerable amount of beneficial substances in beetroot pomace.

Black chokeberry is among the richest sources of anthocyanins responsible for various health-beneficial properties. The majority of chokeberries are used for the production of juice with extremely potent antioxidant activity (*8*). Chokeberry fruit is rich in dietary fibre (up to 5.6% of fresh mass) and chokeberry pomace is a good source of cellulose, hemicellulose and lignin (*8*). Among various chokeberry products, including fresh fruit and juice, the highest total phenolic content and anthocyanin content were found in chokeberry pomace, containing skin and seeds (*9*). Therefore, there is a realistic possibility to use chokeberry pomace as a raw material for the isolation of bioactive compounds or as an ingredient of functional food.

Food waste management has become a challenging task for the food processing industry due to a growing concern about environmental issues in recent years as well as the adoption of sustainable development goals (*10*). The large quantity of produced pomace, especially apple pomace, suggests that one route of its utilisation would not resolve the problem entirely. The development of a single product is not economically feasible. Also, diversification of products based on pomace from the juice industry would lead to better exploitation of underutilised sources of valuable phytochemicals. It is, therefore, worthwhile to explore the production of alcoholic beverages using pomace as raw material.

According to epidemiological studies, the impact of moderate consumption of alcoholic beverages on lipid metabolism and the prevention of coronary artery diseases and colon cancer is related to polyphenol compounds and antioxidant activity (*11*). Rodríguez Madrera *et al.* (*12*) produced a spirit with an alcoholic strength of 60% from dry apple pomace and selected yeast strains, whereas Zhang *et al.* (*13*) evaluated the influence of pectinase treatment on fruit spirits produced from apple mash, juice and pomace. An increasing trend in the development of new fruit-based liquors was already noted by Santos *et al.* (*14*), but according to our knowledge, the possibility of using pomace in the production of liqueurs with a high phenolic content and antioxidant activity has not been investigated yet. This study seeks to address this gap.

The main aim of this research is to examine the possibility of application of apple, beetroot and chokeberry pomace, both individually or in combination, in liqueur production. In that regard, sugar content, non-volatile and volatile acidity, pH and turbidity were analysed in the obtained liqueurs. Sensory quality and consumer acceptability of the freshly prepared liqueurs were also evaluated. Additionally, the composition of individual phenolic compounds in fresh products was assessed. Changes in total phenolic content, antioxidant activity and chromatic characteristics of freshly prepared liqueurs were followed during six months of storage at refrigeration and room temperature (4 and (20±2) °C respectively) to provide preliminary insight in the produced liqueur stability during storage and to elucidate appropriate storage conditions that would ensure good retention of phenolics as bioactive compounds responsible for beneficial health effects and preservation of colour, as an important aspect for the acceptance of novel products.

## MATERIALS AND METHODS

### Chemicals and raw materials

Folin-Ciocalteau reagent, sodium carbonate, sodium acetate trihydrate, acetic acid, hydrochloric acid, sodium hydroxide and phenolphthalein were obtained from Merck (Darmstadt, Germany), DPPH (2,2-diphenyl-1-picrylhydrazyl) from Fluka (Buchs, Switzerland), Trolox (6-hydroxy-2,5,7,8-tetramethylchromane-2-carboxylic acid), 2,4,6-tripyridyl-*s*-triazine (TPTZ) and gallic acid from Sigma-Aldrich, Merck (Munich, Germany), ethanol 96% from Ada Vrenje (Belgrade, Serbia), glycerol from Oleohemija (Belgrade, Serbia), while citric acid was purchased on the local market. Glucose, fructose and sucrose were acquired from Tokyo Chemical Industry, TCI (Zwijndrecht, Belgium). All aqueous solutions were prepared using ultrapure water (Thermo Fisher TKA MicroPure water purification system, *ϰ*=0.055 µS/cm; Thermo Fisher Scientific, Bremen, Germany). Phenolic standards (protocatechuic, *p*-hydroxybenzoic, ellagic, chlorogenic, caffeic, *p*-coumaric, ferulic and sinapic acids, rutin, naringin, pterostilbene, aesculin, quercetin, quercetin-3-O-rhamnoside, quercetin-3-O-galactoside, isorhamnetin and isorhamnetin-3-O-rutinoside) were supplied by Sigma-Aldrich, Merck (Steinheim, Germany).

The company Healthy Organic (Selenča, Serbia) provided apple and beetroot pomace, while chokeberry pomace was acquired from the family farm of D. M. Perić (Belgrade, Serbia). Wet pomace, collected immediately after juice production, was dried at the industrial scale level at 55 °C using the dehydrator Solaris (NTIM Tehnology, Belgrade, Serbia) and ground in an industrial mill to produce a fine powder that is easy to preserve, store, transport and use as a food ingredient (*15*, *16*).

### Liqueur preparation

For the preparation of the liqueurs, a traditional procedure involving a pilot-scale maceration was used, including two blending tanks with agitation systems (30 L), located at the experimental farm Radmilovac (Faculty of Agriculture, University of Belgrade, Serbia). In the first tank, powdered pomace was macerated in a water-alcohol mixture (*φ*(alcohol)=40%) for 24 h at (20±2) °C. The pomace to solvent ratio was 1:10 (*m*/*V*). Extracts obtained after decanting and filtration through disc filters Fibrafix AF31H (12-5.0 μm retention rate; Filtrox AG, St. Gallen, Switzerland) were transferred into the second tank and commercial sugar (Crvenka a.d., Crvenka, Serbia) and water were added to obtain 150 g/L sugar and a final *φ*(alcohol)=20%. Citric acid (1 g/L) was added to achieve a sweetness-sourness balance and glycerol (2 mL/L) was used as a body enhancer. Apple pomace liqueur, chokeberry pomace liqueur, a mixture of the two in a 50:50 ratio and a combination of beetroot pomace liqueur with apple pomace liqueur in the 30:70 ratio were formulated. Beetroot was used only in combination to mitigate its pronounced, undesirable earthy flavour.

### Physicochemical properties of fresh pomace liqueurs

The turbidity of the analysed liqueurs was determined with a portable turbidimeter (model 2100Q; Hach, Loveland, CO, USA). The results of turbidity measurement are expressed as formazin turbidity with a reading range between 0 and 1000 nephelometric turbidity units (NTU). Non-volatile and volatile acidity (g/L) were determined according to AOAC method 945.08 (*17*), while the pH was measured by WTW Multi 9310 apparatus (WTW, Weilheim, Germany).

### Determination of sugars in fresh pomace liqueurs

The liqueurs were filtered through 0.22-µm filter and the filtrate was analysed using the HPAEC-PAD technique on an ISC 3000 DP liquid chromatograph (Dionex, Sunnyvale, CA, USA) equipped with a quaternary gradient pump (Dionex, Sunnyvale, CA, USA) according to the procedure reported by Vasić *et al*. (*18*). The total amount of each sugar compound was calculated from the corresponding calibration curve and expressed in g/L. The linear range was 0.01-0.1 g/L with correlation coefficients over 0.998. The recovery was between 92 and 108%. The limit of detection was from 0.0012 to 0.0034 g/L and the limit of quantification between 0.004 and 0.01 g/L. The precision was lower than 3% and accuracy was around 97%.

### Sensory quality rating of fresh pomace liqueurs

The sensory quality of the freshly prepared liqueur samples was assessed in the sensory testing laboratory by a 6-member panel (35-60 years old; 4 male and 2 female) consisting of staff members from the Faculty of Agriculture, University of Belgrade, Serbia, experienced in alcoholic beverage quality judging. The samples were labelled with random 3-digit codes and presented to the panellists monadically in random order. Low sodium bottled water was used for palate cleansing. Overall sensory quality was assessed by evaluating five selected sensory characteristics: colour, clarity, distinction, odour (orthonasal olfaction) and flavour, which were rated using category scales with score ranges 0-1, 0-1, 0-2, 0-6 and 0-10, respectively. The quality of the beverages was rated as follows: excellent quality (quality score>18), very good quality (16-18), good quality (14-16), poor/unsatisfactory quality (12-14) and very poor quality (score≤12). The overall quality score, with a maximum value of 20, was calculated by adding the quality scores of the five individual characteristics. The panel evaluated all of the samples once.

### Consumer sensory testing of pomace fresh liqueurs

Consumer acceptance tests were performed in a sensory testing laboratory by 143 students (21-25 years old; 92 female and 51 male) from the Faculty of Agriculture, University of Belgrade. The students were randomly selected, provided that they were relatively frequent (at least occasional) consumers of alcoholic beverages. The samples were labelled with random 3-digit codes and presented to the consumer panel monadically in random order. Low sodium bottled water was used for palate cleansing. Overall acceptance, appearance, odour and flavour acceptance were assessed using the 9-point hedonic scale (1=dislike extremely, 5=neither like nor dislike, 9=like extremely). The just-about-right (JAR) scales (1=too little, 5=JAR, 9=too much) were used to evaluate the intensities of colour (too light/pale – JAR – too dark), sweetness (not sweet enough – JAR – too sweet), sourness (not sour enough – JAR – too sour) and alcoholic strength (too weak – JAR – too strong). In addition, 9-point attribute intensity scales were used to assess consumer perception of fullness of flavour (1=empty, 5=medium, 9=full) and distinctiveness of flavour (1=not at all, 5=medium, 9=completely characteristic).

### Identification and quantification of phenolics in fresh pomace liqueurs by HPLC–DAD–MS/MS

After filtration of samples through a 0.22-µm filter, individual phenolic compounds were identified and quantified in the filtrate using a Dionex Ultimate 3000 UHPLC system equipped with a diode array detector connected to a TSQ Quantum Access Max triple quadrupole mass spectrometer (Thermo Fisher Scientific, Basel, Switzerland) with the ion source in the form of electrospray ionisation (200 °C) in the negative mode (from *m*/*z*=100 to 1000) using triple quadrupole (UHPLC-DAD-MS/MS), according to previously published procedure (*19*). The total amount of each compound was calculated from the corresponding calibration curve and expressed in mg/L. The linear range was 0.001-0.1 g/L, whereas the correlation coefficients were from 0.9945 to 0.9996. The recovery of the method was between 85 and 115%. The limit of detection was from 0.00001 to 0.00012 g/L, whereas the limit of quantification was in the range of 0.00005-0.00021 g/L. The precision was less than 5% and accuracy was in the range of 91 to 105%.

### Determination of total phenolic concentration and antioxidant activity in fresh and stored pomace liqueurs

The total phenolic concentration of the prepared liqueurs was determined by the Folin-Ciocalteu method described by Singleton *et al.* (*20*). Properly diluted samples (0.5 mL) were mixed with 0.1 M Folin-Ciocalteu reagent (2.5 mL), then 2.5 mL sodium carbonate solution (75 g/L) were added after 6 min in the dark, the mixture was left for 2 h in the dark, after which the absorbance at 740 nm was measured using spectrophotometer (Thermo Scientific Evolution 600; Thermo Fisher Scientific Inc., Bremen, Germany), using distilled water as a blank. The results were expressed in gallic acid equivalents (GAE) per litre of liqueur. The antioxidant capacity was determined by DPPH and ferric reducing antioxidant power (FRAP) assays, using procedures described by Blois (*21*) and Benzie and Strain (*22*) respectively. Diluted samples (0.2 mL) were mixed with 2.8 mL ethanolic solution of DPPH (0.1 mM) and acetate buffer (0.1 M) in the volume ratio 2:1, and the mixture was allowed to react for 30 min in the dark before absorbance measurement at 517 nm against distilled water (Thermo Scientific Evolution 600; Thermo Fisher Scientific Inc). Diluted samples (0.1 mL) were mixed with distilled water (0.3 mL) and freshly made FRAP reagent (3 mL), incubated for 40 min at 37 °C and the absorbance was measured against the reagent blank at 593 nm. The results of DPPH and FRAP were expressed in mM Trolox equivalent (TE) per litre of the sample. Measurements were performed on the first day (no storage) and upon each month during six months of liqueur storage at (20±2) °C in a dark place and in a refrigerator (4 °C). All measurements were performed in triplicate.

### Colour measurements in fresh and stored pomace liqueurs

Colour intensity (CI) and hue (*h*) were determined according to Glories (*23*). Liqueurs were diluted to 1:10 with 20% ethanol, centrifuged for 5 min at 3000×*g* using centrifuge (Tehtnica, Železniki, Slovenia) and absorbance was measured at 420, 520 and 620 nm in a 1-cm cell path using a spectrophotometer (Thermo Scientific Evolution 600; Thermo Fisher Scientific Inc). CI was calculated as:

CI=*A*_420 nm_+*A*_520 nm_+*A*_620 nm_ /1/

whereas hue (*h*) was calculated as:

*h*=*A*_420 nm_/*A*_520 nm_ /2/

Measurements were performed on the first day (no storage) and upon each month during six months of liqueur storage at (20±2) °C in a dark place and in a refrigerator (4 °C).

### Statistical analysis

The total phenolic content, antioxidant capacity, colour intensity and hue were measured in triplicate and the results are presented as mean value±standard deviation (S.D.). The data related to total phenolic content, antiradical activity (DPPH), total reducing power (FRAP), and analytical colour measurements (colour intensity and hue) were subjected to principal component analysis (PCA). PCA was performed on the unfolded data matrix which included all replicate measurements. Upon dimension reduction, when it was clear that the first extracted principal component (PC1) was sufficient enough to satisfactorily explain the variations in the data matrix, PC1 scores for samples were subjected to 3-way ANOVA (PC-ANOVA) (*24*) with product/pomace, storage time and storage temperature taken as fixed factors. Also, another PC-ANOVA model, followed by Tukey’s honestly significant difference (HSD) test, was applied in order to separate the mean PC1 scores for samples.

Sensory quality and acceptance (hedonic and attribute intensity) data were subjected to 2-way ANOVA with samples as a fixed factor, and assessors as a random factor. Tukey's HSD test was used to separate the mean values of samples.

Mean drop analysis was performed by combining the JAR data with the overall hedonic data, as described by Rothman and Parker (*25*), in order to assess the potential impact of being off from just-about-right on the overall acceptability of the liqueurs. Raw JAR scores were grouped into three categories as follows: 1, 2 and 3=below JAR; 4, 5 and 6=JAR; and 7, 8 and 9=above JAR. Mean drop values were calculated by subtracting the overall mean hedonic scores of each below/above JAR category from the hedonic mean of the JAR category. ANOVA and Tukey's HSD test were used in order to compare the overall hedonic mean values of the JAR and non-JAR categories. Minimum percentage skew for ‘not just right’ (the cut-off) was set at 20% of the total consumer panel.

Statistical analyses were performed using SPSS Statistics v. 17.0 (*26*). The level of statistical significance was set at 0.05.

## RESULTS AND DISCUSSION

### Physicochemical properties and sugar content of fresh pomace liqueurs

The results of physicochemical analysis (turbidity, pH, non-volatile and volatile acidity) and quantitative sugar profile of liqueurs are summarized in Table 1. The highest value of turbidity was determined in the apple/beetroot pomace liqueur (250.4 NTU), followed by apple pomace liqueur (240.0 NTU) and apple/chokeberry pomace liqueur (229.8 NTU), whereas the lowest value was ascribed to chokeberry pomace liqueur (102.6 NTU). Apple pomace is a rich source of compounds with colloidal properties (*i.e.* dietary fibre), so as expected, liqueurs produced with this raw material showed higher turbidity (*27*).

**Table 1:** Physicochemical parameters and sugar mass concentrations of fresh liqueurs produced from apple, chokeberry and beetroot pomace

Parameter	AL	CL	ACL	ABL
Turbidimetry/NTU	240.0±1.0	102.6±0.9	229.8±4.4	250.4±2.1
pH	3.30±0.02	3.60±0.02	3.50±0.02	3.72±0.02
Non-volatile acidity as *γ*(malic acid)/(g/L)	2.48±0.01	2.84±0.03	2.64±0.01	2.80±0.01
Volatile acidity as *γ*(acetic acid)/(g/L)	0.32±0.02	0.33±0.03	0.32±0.02	0.29±0.02
*γ*(glucose)/(g/L)	(8±1)^a^	(31±3)^b^	(21±2)^c^	(13±1)^d^
*γ*(fructose)/(g/L)	(16±2)^a^	(24±2)^b^	(22±2)^bc^	(19±2)^ac^
*γ*(sucrose)/(g/L)	(195±18)^a^	(148±11)^b^	(159±13)^a^	(177±16)^a^
*γ*(total sugar)/(g/L)	(219±17)^a^	(203±11)^a^	(202±16)^a^	(208±16)^a^

L=liqueur, A=apple, C=chokeberry, B=beetroot. Values with the same letter in superscript within the same row are not statistically different (α=0.05)

Herein, the obtained pH values of prepared liqueurs were between 3.30 (apple pomace liqueur) and 3.72 (apple/beetroot pomace liqueur). Corroborating the obtained results, the pH values of differently prepared apple wines, reported by Won *et al*. (*28*), were lower than 4. Also, pH values of a variety of chokeberry products ranged from 3.31 to 4.28 (*29*). The pH value of apple/beetroot liqueur (3.72) was similar to the pH of beetroot-based wines (3.56-4.00) reported by Singh *et al*. (*30*) and (3.45-3.80) reported by Soibam *et al.* (*31*).

No marked difference was evident in the obtained values for non-volatile and volatile acids in all analysed liqueurs. The mass concentration of non-volatile malic acid ranged from 2.48 to 2.84 g/L, whereas the mass concentration of volatile acetic acid was from 0.16 to 0.33 g/L. These results are in line with the values for the total acidity of apple liqueurs (1.16-5.82 g/L) reported by Díez Marqués *et al*. (*32*). Liqueurs are alcoholic beverages produced without fermentation, so, expectedly, the volatile acidity is low. On the other hand, acidity regulators such as citric acid, added in the tested samples, in addition to malic and citric acids originating from the apple, also contribute to the pronounced total acidity (*32*).

As expected, the most abundant sugars in freshly prepared liqueurs detected by HPAEC-PAD technique were glucose, fructose and sucrose. Due to the significant amount of added sugar (150 g/L), the concentration of sucrose was expectedly the highest in all samples when compared to glucose and fructose. As shown in Table 1, there is no significant difference in the total sugar concentration among all analysed liqueurs, which can be explained by the different ratios of individual sugars (glucose, fructose and sucrose) in apple, chokeberry and beetroot pomace. Indeed, the absence of sucrose in cultivated black chokeberries is an important characteristic of its sugar profile (*33*), while beetroot is a valuable source of sucrose and a scarce source of glucose and fructose (*34*). In the case of the apple-based samples (apple, apple with chokeberry and apple/beetroot pomace liqueurs), the concentration of fructose was higher than glucose, which is in agreement with a previous study (*35*). The fresh chokeberry fruit contains a slightly higher concentration of glucose than fructose (*8*), which is also found in the liqueur prepared with chokeberry pomace.

### Sensory properties of fresh pomace liqueurs

According to the results of the sensory quality rating of the liqueurs (Table 2), it seems that selected sensory characteristics were rated in a similar way over the spectrum of the evaluated products. The mean odour quality score for apple/beetroot pomace liqueur (4.4±0.5) was significantly lower (p<0.05) than the other three liqueurs placed within the same homogenous subset. The main defects regarding its odour were the undesirable aroma and flavour linked to an earthy note known to be caused by the volatile bicyclic alcohol geosmin (*trans*-1,10-dimethyl-*trans*-9-decalol) (*36*). The lowest mean overall sensory quality score was obtained for the apple/beetroot liqueur (16.6±0.7), noting that the value differed significantly (p<0.05) from the chokeberry only liqueur, which was the best rated (17.6±0.9). The uniqueness of chokeberry liqueur can be explained by the sensory experts’ additional notes that its flavour and odour were characterised by an appealing and desirable sour cherry aroma. Regardless of the difference, all mean overall quality scores (16.6-17.6) were in the range of ‘very good quality’.

**Table 2:** Sensory quality scores for the fresh liqueurs produced from apple, chokeberry and beetroot pomace

Sample	Colour* (max. 1)	Clarity* (max. 1)	Distinction* (max. 2)	Odour** (max. 6)	Flavour** (max. 10)	Overall score** (max. 20)
AL	1	1	2	(5.3±0.6)^b^	8.0±0.4	(17.3±0.9)^ab^
CL	1	1	2	(5.3±0.1)^b^	8.4±0.3	(17.6±0.3)^b^
ABL	1	1	2	(4.4±0.5)^a^	8.2±0.2	(16.6±0.7)^a^
ACL	1	1	2	(5.3±0.5)^b^	8.4±0.5	(17.6±0.9)^ab^
L=liqueur, A=apple, C=chokeberry, B=beetroot. *Values are modes (6 assessors, 1 repetition), **values are arithmetic mean±standard deviation (6 assessors, 1 repetition). Values marked with the same letter under the same type of spirit are not statistically different (α=0.05)						

**Fig. 1:**
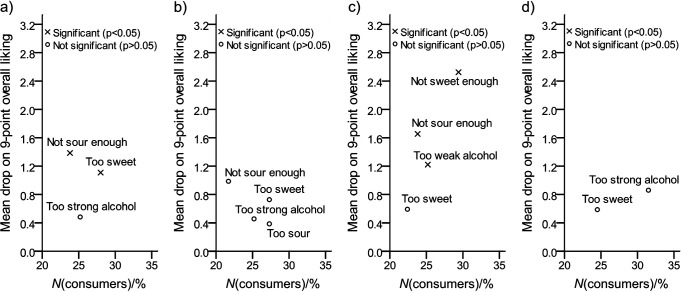
Mean drop analysis (*N*(consumer)=143) for the freshly prepared liqueur samples. A circle in the plot that shows statistically significant mean drop and a large percentage of consumers (above 20% in this case) is a cause for concern and suggests that the product should be modified in the appropriate direction. Liqueurs from the pomace of: a) apple, b) chokeberry, c) apple and beetroot (70:30) and d) apple and chokeberry (50:50), mean drop=the drop of the mean hedonic score calculated as the difference between the ’just-about-right’ consumer group and the ’too much’ or ’not enough of an attribute’ consumer groups

The results of testing the likeability of the liqueurs are shown in Table 3. The overall acceptance, odour and flavour mean hedonic scores of apple/beetroot liqueur (4.5, 4.5 and 4.3, respectively) were in the range of neutral consumer attitude, *i.e.* ’neither like nor dislike‘ and were significantly lower (p<0.05) than the scores of the other liqueurs that were found in the range of ‘liking’ (≥6.0). On the other hand, by using a 9-point attribute intensity scale, consumers perceived ‘fullness’ and ‘distinctiveness’ of apple/beetroot liqueur flavour at the same intensity level (p>0.05). These results, together with the results of sensory quality testing, indicate that lower hedonic scores for this liqueur were not the result of a lack of flavour, but can most probably be directly linked to the acceptability of the typical earthy, leafy and neutral flavour of beetroot in alcoholic spirits. This conclusion is also supported by the results of mean drop analysis (Fig. 1). There were three large groups of results among consumers (≥20%) for apple/beetroot liqueur tested. Significant mean drops of the overall hedonic scores (p<0.05) were observable, with the opinion that the product was ‘not sweet enough’ (29.4%), ‘not sour enough’ (23.8%), or it was ‘too weak’ (25.2%) in terms of alcohol level. When compared to the other liqueurs, no large consumer groups with significant mean drops were observed for the chokeberry pomace liqueur, whereas for the apple pomace liqueur sample, it can be seen that consumers complained that the product was ‘too sweet’ and ‘not sour enough’.

**Table 3:** Sensory acceptance of the fresh liqueurs produced from apple, chokeberry and beetroot pomace

Sample	Overall acceptance*	Appearance acceptance*	Odour acceptance*	Flavour acceptance*	Fullness of flavour**		Distinctiveness of flavour**
AL	(6.2±2.2)^b^	(7.0±1.9)^b^	(6.6±2.0)^c^	(6.0±2.3)^b^	6.2±1.9	6.2±2.3		
CL	(6.1±2.3)^b^	(7.4±1.9)^b^	(6.0±2.3)^b^	(6.0±2.4)^b^	6.3±1.9	6.4±2.0		
ABL	(4.5±2.5)^a^	(6.3±2.1)^a^	(4.5±2.6)^a^	(4.3±2.7)^a^	5.9±2.2	6.7±2.3		
ACL	(6.3±2.4)^b^	(7.0±1.9)^b^	(5.9±2.3)^b^	(6.2±2.4)^b^	6.4±2.0	5.9±2.3		
L=liqueur, A=apple, C=chokeberry, B=beetroot. *Ratings on the 9-point hedonic scale, **ratings on the 9-point attribute intensity scale (a consumer concept).Values are arithmetic mean±standard deviation (*N*=143). Values marked with the same letter within the same column are not statistically different (α=0.05)								

### The phenolic profile of fresh pomace liqueurs

The mass concentration of individual phenolics in liqueurs is shown in Table 4. Chokeberry-based liqueurs (chokeberry and apple/chokeberry liqueurs) seem to be the richest source of tested phenolics. In the study of Sokoł-Łȩtowska *et al.* (*37*) the amount of phenolic compounds in chokeberry liqueur was also predominant compared to cornelian cherry, black rose, blackcurrant, blackberry, raspberry, mahonia, sloe, strawberry and sour cherry liqueurs. The predominance of ellagic acid in chokeberry, apple/chokeberry and apple/beetroot liqueurs can be easily observed, with chokeberry and apple/chokeberry liqueurs containing by far superior concentrations. A high mass concentration of phlorizin was evident in all liqueurs containing apple pomace, as expected since it was suggested to be used as an apple pomace marker (*19*). Quercetin and its sugar derivatives were also present in significant amounts in all liqueurs, with chokeberry and apple/chokeberry liqueurs being the most endowed. According to previous studies, quercetin was the predominant flavonol in chokeberry (*38*). Also, chokeberry wine contained quercetin as the most abundant flavonoid, and represented the richest source of this flavonol, as well, when compared to some fruit wines such as blackberry wine, sour cherry wine, *etc.* (*39*). Corroborating the results obtained for apple pomace liqueur, quercetin was one of the major flavonols in apple pomace from several cultivars in which the presence of quercetin glycosides was observed, as well as the prevalence of galactoside over rhamnoside (*19*). Other phenolic compounds found in liqueurs in notable amounts are ferulic acid, 5-*o*-caffeoylquinic acid, protocatechuic acid, phloretin, *p*-hydroxybenzoic acid, rutin, *p-*coumaric acid and pterostilbene.

**Table 4:** Phenolic profile of the fresh liqueurs produced from apple, chokeberry and beetroot pomace

Phenolic compound	AL	CL	ACL	ABL
*γ*/(mg/L)			
Ellagic acid	34.8±0.3	293±7	191.2±6.0	128.3±0.1
Phlorizin	93.26±0.04	n.d.	62.3±0.9	51.83±0.04
Phloretin	10.12±0.03	n.d.	5.33±0.01	4.04±0.00
Quercetin	10.5±0.0	19.56±0.01	16.79±0.04	14.64±0.04
Quercetin-3-O-galactoside	7.66±0.03	9.97±0.09	9.6±0.1	8.85±0.02
Quercetin-3-O-rhamnoside	4.72±0.04	4.79±0.06	4.21±0.05	3.76±0.03
Ferulic acid	11.46±0.08	12.4±0.1	12.0±0.4	8.63±0.04
5-O-caffeoylquinic acid	12.39±0.07	11.36±0.05	11.2±0.1	3.46±0.05
Protocatechuic acid	4.95±0.04	7.1±0.1	7.05±0.07	4.35±0.01
*p*-Hydroxybenzoic acid	5.37±0.07	3.75±0.01	3.67±0.07	0.023±0.00
Rutin	2.81±0.04	4.2±0.1	3.13±0.04	3.35±0.05
*p*-Coumaric acid	1.94±0.03	4.09±0.06	3.98±0.06	4.35±0.05
Pterostilbene	1.44±0.00	1.6±0.1	1.2±0.1	0.23±0.00
Aesculin	0.86±0.03	0.69±0.03	0.76±0.05	0.74±0.05
Isorhamnetin-3-O-rutinoside	0.66±0.02	0.86±0.03	0.74±0.02	0.60±0.03
Isorhamnetin	0.38±0.00	0.53±0.00	0.45±0.02	0.43±0.04
Caffeic acid	0.26±0.01	0.63±0.02	0.62±0.03	0.01±0.00
Naringin	0.36±0.00	0.38±0.00	0.37±0.01	0.46±0.02
Sinapic acid	0.23±0.01	0.25±0.01	0.24±0.01	0.11±0.00
Taxifolin	0.26±0.01	0.27±0.01	0.29±0.00	0.15±0.00
L=liqueur, A=apple, C=chokeberry, B=beetroot, n.d.=not determined				

### Total phenolic content and antioxidant capacity of fresh pomace liqueurs

All produced liqueurs showed notable total phenolic content at the time of preparation, with the following descending order of activities: chokeberry>apple/chokeberry>apple>apple/beetroot liqueur (Table 5). Total phenolic concentration (TPC) for chokeberry liqueur (expressed in gallic acid equivalents (GAE) of (3473±33) mg/L) was in line with the previous findings of Sokoł-Łȩtowska *et al.* (*37*), who showed chokeberry liqueur to be among the richest sources of substances reacting with the Folin-Ciocalteu reagent (3292 mg/L). The total phenolic concentration of sour cherry liquor was reported at comparable value (3360 mg/L (*40*)). In comparison with the total phenolic concentration of commercial Portuguese Terras Madeirenses red wines ((1724-1871) mg/L) (*41*), chokeberry and apple/chokeberry liqueurs showed almost double concentration, as well as far higher concentrations than those of various red and white wines from the Serbian market (164 to 2314 mg/L) (*42*). Similarly, when compared to the herbal bitter liqueur based on medicinal plant extracts (*43*) that contained a conclusively larger concentrations of phenolics (1500 mg/L) than similar commercial herbal spirits, chokeberry and apple/chokeberry liqueurs showed twice as high total phenolic concentration, as well as several times stronger antioxidant activity measured by DPPH and FRAP assays.

**Table 5:** Changes in total phenolic content and antioxidant activity determined by DPPH and FRAP occurring during 6 months of storage of apple, chokeberry and beetroot pomace liqueurs at two different temperatures

Temperature/°C	*t*/month		AL					CL				ACL			ABL				
*ɣ*(TPC as GAE)/(mg/L)	*c*(DPPH)/(mmol/L)		*c(*FRAP)/(mmol/L)		*ɣ*(TPC as GAE)/(mg/L)	*c*(DPPH)/(mmol/L)		*c(*FRAP)/(mmol/L)	*ɣ*(TPC as GAE)/(mg/L)	*c*(DPPH)/(mmol/L)	*c(*FRAP)/(mmol/L)	*ɣ*(TPC as GAE)/(mg/L)			*c*(DPPH)/(mmol/L)	*c(*FRAP)/(mmol/L)
no storage			(871±32)^f^	(3.1±0.0)^h^	(8.1±0.1)^h^		(3473±33)^i^			(28.0±0.7)^h^	(58.9±0.5)^i^	(2960±35)^h^	(16.2±0.3)^h^	(29.5±1.3)^e^		(790±8)^g^	(2.3±0.2)^f^		(9.6±0.4)^g^
4		1	(646±24)^e^	(2.8±0.0)^fg^	(8.5±0.1)^h^		(3205±42)^g^			(21.9±0.4)^g^	(51.5±1.3)^g^	(2177±54)^g^	(12.9±0.39)^g^	(31.6±0.4)^f^		(524±18)^f^	(1.9±0.0)^e^		(5.8±0.1)^e^
		2	(645±9)^e^	(2.6±0.1)^f^	(7.7±0.3)^g^		(3218±21)^g^			(19.3±0.6)^f^	(47.9±0.4)^f^	(1977±18)^ef^	(11.34±0.2)^d^	(28.4±1.3)^de^		(482±4)^e^	(1.6±0.1)^d^		(5.0±0.1)^d^
		3	(552±6)^d^	(2.0±0.2)^e^	(5.4±0.1)^e^		(29429±11)^f^			(19.6±0.2)^e^	(35.1±0.6)^e^	(1919±47)^e^	(12.7±0.2)^fg^	(23.2±0.5)^c^		(358±4)^d^	(1.2±0.0)^c^		(3.6±0.1)^c^
		4	(409±3)^c^	(1.5±0.1)^d^	(4.0±0.1)^d^		(2658±26)^e^			(16.3±0.7)^b^	(27.3±0.2)^b^	(1579±18)^c^	(8.7±0.8)^c^	(17.6±0.1)^b^		(321±1)^c^	(0.8±0.1)^b^		(3.0±0.0)^b^
		5	(256±2)^ab^	(0.9±0.0)^ab^	(2.9±.0.0)^c^		(2500±20)^d^			(17.6±0.6)^cde^	(31.8±0.2)^cd^	(840±17)^a^	(4.8±0.2)^a^	(9.8±0.4)^a^		(139.0±2.6)^a^	(0.7±0.2)^b^		(1.2±0.1)^a^
		6	(226±2)^a^	(1.2±0.0)^bc^	(2.3±0.1)^b^		(1729±15)^a^			(14.4±0.3)^a^	(19.9±0.9)^a^	(784±13)^a^	(7.9±0.4)^c^	(10.3±0.1)^a^		(153.1±2.1)^a^	(0.6±0.1)^ab^		(1.3±0.1)^a^
20		1	(640±2)^e^	(2.9±0.0)^gh^	(7.6±0.1)^g^		(3302±33)^h^			(23.8±0.5)^h^	(54.3±0.7)^h^	(2228±58)^g^	(11.5±0.2)^d^	(28.1±0.8)^de^		(527±4)^f^	(2.0±0.1)^e^		(6.5±0.1)^f^
		2	(618±6)^e^	(2.2±0.0)^e^	(6.6±0.1)^f^		(3400±10)^i^			(21.9±0.2)^g^	(51.8±0.8)^g^	(2035±13)^ef^	(11.8±0.5)^df^	(26.2±1.5)^d^		(484±3)^e^	(1.6±0.0)^d^		(5.0±0.0)^d^
		3	(542±2)^d^	(2.2±0.1)^e^	(3.4±0.1)^d^		(2996±26)^f^			(21.0±0.5)^de^	(33.3±0.8)^de^	(1749±33)^de^	(11.7±0.3)^df^	(16.3±0.3)^b^		(317±1)^c^	(1.3±0.0)^c^		(3.4±0.1)^bc^
		4	(424±3)^c^	(1.4±0.2)^cd^	(3.3±0.5)^d^		(2648±29)^e^			(18.5±0.5)^c^	(30.2±0.2)^c^	(1280±52)^b^	(6.1±0.1)^b^	(16.3±0.6)^b^		(332±5)^c^	(0.4±0.1)^a^		(3.3±0.5)^bc^
		5	(255±4)^ab^	(0.8±0.1)^ef^	(1.3±0.1)^ab^		(2034±21)^c^			(14.5±0.8)^a^	(21.5±0.4)^a^	(832±42)^a^	(4.8±0.0)^a^	(8.2±0.5)^a^		(175±4)^b^	(0.5±0.0)^ab^		(1.3±0.1)^a^
		6	(268±6)^b^	(0.9±0.0)^a^	(1.3±0.1)^a^		(1718±71)^a^			(14.6±0.2)^a^	(21.2±0.9)^a^	(874±9)^a^	(5.9±0.1)^b^	(8.0±0.3)^a^		(181±4)^b^	(0.5±0.0)^ab^		(1.29±0.1)^a^
L=liqueur, A=apple, C=chokeberry, B=beetroot; TPC= total phenolic concentration expressed as gallic acid equivalent (GAE) per litre of liqueur. Antioxidant activity (DPPH and FRAP) expressed as Trolox equivalent per litre of liqueur. Values are arithmetic mean±standard deviation. Values marked with the same letter within the same column are not statistically different (α=0.05)																			

It can be noticed that the total phenolic concentration determined by Folin-Ciocalteu assay was higher than the sum of individual phenol concentrations quantified by HPLC. This is in line with previous studies that explained such result by the interference of various substances other than phenols (organic acids, residual sugars, amino acids, proteins and other hydrophilic compounds) in the Folin-Ciocalteu assay, various responses of individual phenols, presence of only low molecular mass phenols in extracts (*5*, *19*), as well as missing values of unidentified polyphenols by HPLC/MS.

A similar antioxidant activity of liqueurs measured by DPPH and FRAP assays was obtained as for total phenolic concentration, with chokeberry liqueur being by far the strongest radical scavenger ((28.0±0.7) and (58.9±0.5) mmol/L, respectively). However, in the case of results obtained by FRAP assay, it can be observed that apple/beetroot liqueur had slightly higher antioxidant potential than apple liqueur.

### Preliminary evaluation of liqueur storability based on changes of total phenolics and antioxidant activity

The results of total phenolic concentration and antioxidant capacity trends of analysed liqueurs during six months of storage at two different temperatures are presented in Table 5. As evident, total phenolic concentration and antioxidant activity decreased during storage at 20 and 4 °C. However, the decrease differs among liqueurs. After six months, the amount of initial total phenolic content of apple, chokeberry, apple/chokeberry and apple/beetroot liqueurs stored at 4 and 20 °C decreased by about 75, 50, 70 and 80%, and by 70, 50, 70 and 77%, respectively. Throughout the entire storage period, the highest retention of phenolics was observed in chokeberry liqueur, which preserved a much higher concentration at the end of storage than the initial total phenolic concentration of apple and apple/beetroot liqueurs.

In all cases, with the exception of chokeberry liqueur stored at 4 °C, there were no significant differences in total phenolic concentrations after 5 and 6 months of storage, leading to the assumption that the decomposition of phenolic compounds is complete after 5 months.

The decrease of antioxidant activity of chokeberry and apple/chokeberry liqueurs during storage, measured by DPPH, was also the least prominent (by approx. 50%) compared to apple and apple/beetroot liqueur, where drops greater than 60% were observed. At the same time, antioxidant capacity reduction determined by FRAP method was between 65-85% for all analysed liqueurs.

There is scarce literature data on the possibility of utilisation of apple, beetroot and chokeberry pomace in the production of antioxidant-rich alcoholic or non-alcoholic beverages. In a study dealing with antioxidant activity of liqueurs made from ten red fruits, in the majority of samples, the concentration of phenolic compounds decreased over the considered periods (*37*). The same study demonstrated that chokeberry liqueur was among those with the highest phenolic concentration and antioxidant activity, and when stored at a temperature of 30 °C for 6 months it showed a significant reduction in activity (assayed with the DPPH test) of approx. 50% of the initial value (*37*). Walkowiak-Tomczak (*44*) reported that after 20 days under facultative anaerobic conditions, the antioxidant activity of black chokeberry juice concentrate solutions decreased by 7-12% at 10 °C, 12-15% at 20 °C and 16-35% at 30 °C, whereas under aerobic conditions the changes ranged from 63 to 76% after 10 days and from 64 to 79% after 20 days. Furthermore, phenolic compounds in myrtle liqueur showed considerable changes even when stored with constant headspace. The anthocyanins in particular, both total and free, tended to decrease (*45*).

The majority of spirits, including liqueurs, are commonly stored safely at room temperature since alcohol provides microbiological stability. Studies on a half-year period of sour cherry liqueur storage showed that their characteristic features are almost unchanged if stored at 15 °C and without sugar added, but organoleptic properties were better in samples stored at 30 °C (*46*). Here, different storage temperatures did not have a significant influence on the total phenolic concentration of chokeberry and apple/chokeberry liqueurs, but the total phenolic concentration of apple and apple/beetroot liqueurs was significantly higher after 6 months of storage at room temperature than in the refrigerator. It seems that the lower temperature might slightly decrease the solubility of phenolics, inducing their precipitation. However, the differences in the obtained values are not so prominent to enable a conclusion that room temperature is the most appropriate condition for storage of analysed liqueurs.

The strong correlation between total phenolic concentration and antioxidant capacity measured by DPPH and FRAP was confirmed by high correlation coefficients (0.978 and 0.966, respectively). Such a result indicates that the potent antioxidant capacity of the liqueurs is highly influenced by phenolics present in apple, beetroot and chokeberry pomace, as well as in the prepared mixtures, which corroborates the previous reports (*40*, *42*, *47*).

### Preliminary evaluation of liqueur storability based on colour changes

Colour is one of the most important quality features of liqueurs with a huge influence on consumer preferences. The determination of the optimal storage conditions can prevent colour changes that consumers associate with food spoilage and can thus be crucial in preventing economic losses, especially in sales of new products. According to literature, the intense red colour of chokeberry liqueurs depends on the structure and concentration of anthocyanins (*37*). Apple skin colour is influenced by chlorophyll and carotenoids, anthocyanins, flavonols and proanthocyanidins, whereas the beetroot pomace is a rich source of red-coloured betacyanins and yellow pigment betaxanthin (*48*, *49*).

The colour intensity and hue of the analysed liqueurs are shown in Table 6. The significant difference in the chromatic characteristics of liqueurs is primarily due to the type and quantity of pomace pigment compounds.

**Table 6:** Changes of colour intensity (CI) and hue (*h*) values during 6 months of storage of apple, chokeberry and beetroot pomace liqueurs at two different temperatures

Temperature/°C	*t*/month	CI				*h*			
AL	CL	ACL	ABL	AL	CL	ACL	ABL
no storage		0.155^e^	2.259^f^	1.213^h^	0.391^j^	2.784^e^	0.497^a^	0.555^a^	2.924^c^
4	1	0.137^c^	1.058^a^	0.671^e^	0.266^g^	2.871^ef^	0.900^d^	0.954^bc^	2.202^a^
	2	0.170^f^	1.162^d^	0.732^g^	0.327^i^	3.118^fg^	0.869^c^	3.118^g^	2.538^b^
	3	0.094^a^	1.173^d^	0.688^f^	0.127^a^	2.440^d^	0.856^b^	0.877^b^	3.679^f^
	4	0.137^c^	1.104^c^	0.683^f^	0.136^b^	1.391^b^	0.895^d^	0.956^bc^	4.957^i^
	5	0.146^d^	1.173^d^	0.604^d^	0.163^c^	0.679^a^	0.94^e^	0.959^bc^	4.387^h^
	6	0.100^ab^	1.061^a^	0.615^d^	0.161^c^	3.211^g^	0.960^f^	1.023^c^	4.191^gh^
20	1	0.131^c^	1.158^d^	0.607^d^	0.230^f^	2.562^d^	0.996^g^	1.075^cd^	3.118^cd^
	2	0.193^g^	1.224^e^	0.692^f^	0.296^h^	3.118^fg^	1.099^h^	3.118^g^	2.448^ab^
	3	0.100^b^	1.097^bc^	0.511^bf^	0.181^d^	2.636^de^	1.124^i^	1.183^de^	4.052^g^
	4	0.166^f^	1.083^b^	0.490^a^	0.191^e^	1.750^c^	1.186^j^	1.310^ef^	3.265^de^
	5	0.102^bd^	1.107^c^	0.527^c^	0.168^c^	3.277^g^	1.275^k^	1.327^f^	4.451^h^
	6	0.206^h^	1.053^a^	0.520^bc^	0.178^d^	2.656^de^	1.329^l^	1.377^f^	3.548^ef^
L=liqueur, A=apple, C=chokeberry, B=beetroot. Values are arithmetic means (standard deviation values of triplicates were zero or *negligible)*. Values marked with the same letter within the same column are not statistically different (α=0.05); Tukey’s HSD test									

The colour intensity of apple/chokeberry, chokeberry and apple/beetroot liqueurs decreased throughout the evaluated storage period, although the reduction was nonlinear. No particular trend of change in CI over time can be observed for apple liqueur, at both tested temperatures. Comparing the results obtained at the two tested temperatures, it can be observed that the lower temperature did not prevent the degradation of colour during storage.

Except in the case of apple liqueur, an increase in hue was observed during the evaluated storage period, indicating the growth in the percentage of the yellow colour and/or loss of the red colour. The colour of myrtle liqueur, evaluated according to the classic spectrophotometric parameters of intensity and hue, showed marked variability during storage in the bottles with increasing headspace, while values remained almost constant in unopened ones (*45*). The increase in values obtained for the yellow component and hue angle with the ageing time of berry fruit syrup wines with different pH values was previously linked to the formation of anthocyanin-derived yellow-orange pigments like pyranoanthocyanins, as well as to the oxidation of pigments. Also, the red component percentage in these wines decreased after 6 months of storage. The decrease was associated with the possible precipitation of insoluble polymeric anthocyanin-derived pigments, and/or the degradation of free anthocyanins caused by oxidation (*50*).

### Principal component analysis

The results of principal component analysis (PCA) applied to the unfolded data matrix, derived from the antioxidant activity (total phenolic concentration, FRAP and DPPH) and colour measurements (hue and CI), showed that only the first extracted principal component had an eigenvalue larger than one, and according to both the Kaiser criterion and scree plot (*51*), PC1 was retained for describing objects in the new PC space explaining 87.8% of the variance in the data matrix values. All five initial variables had high PC1 loadings, indicating strong correlations of these attributes with PC1. The antioxidant activity variables (total phenolic concentration, FRAP and DPPH), together with colour intensity (CI), showed strong positive correlations (loading values 0.98, 0.96, 0.99 and 0.96, respectively), while hue showed strong negative correlation (-0.79) with PC1. Therefore, taking into account that greater values for total phenolic concentration, FRAP, DPPH and CI (*i.e*. greater positive values of PC1) indicated lower levels of the oxidation processes, PC1 was referred to as ‘antioxidant activity’ axis.

**Fig. 2:**
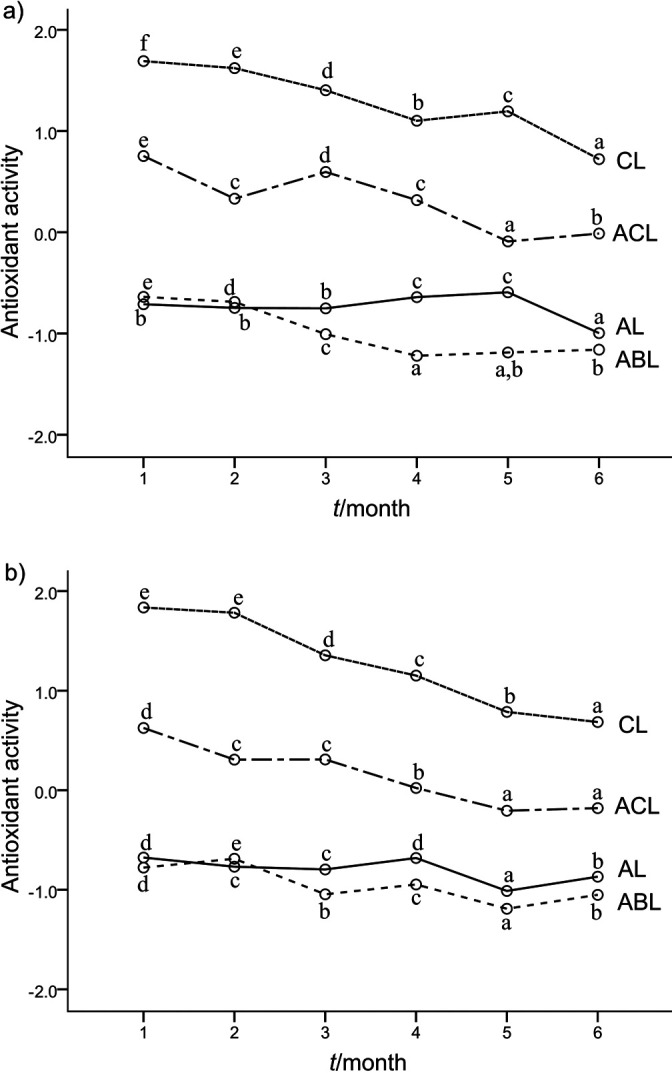
Storage time by type of product/pomace (by storage temperature) interaction profile plots as a result of three-way ANOVA (*N*=3) applied on PC1 scores, for the liqueur samples produced from apple, chokeberry and beetroot pomace stored for six months at: a) 4 °C or b) 20 °C. PCA included the chemical and objective colour variables. PC1 loading values were 0.98, 0.96, 0.99, -0.79 and 0.96 for total phenolic concentrations, FRAP, DPPH, hue and CI, respectively (the combination of these variables was referred to as ’antioxidant activity’). Values marked with the same lower-case letter within a liqueur sample are not statistically different (α=0.05). PC1 scores for control (freshly prepared) samples were 2.65, 1.29, -0.64 and -0.57 for chokeberry (CL), apple and chokeberry (50:50, ACL), apple (AL) and apple and beetroot (70:30, ABL) liqueurs, respectively

The results of ANOVA applied to PC1 scores showed that antioxidant activity was significantly affected (p<0.05) by all examined factors: type of pomace used, storage time and temperature. Also, all interactions among the factors were statistically significant. The plots of factor interactions are shown in Fig. 2. The influence of the type of pomace used for liqueur preparation is consistent over different levels of the storage time factor (chokeberry>apple/chokeberry>apple>apple/beetroot liqueur), indicating a stronger influence of chokeberry pomace on the antioxidant potential of the liqueurs over the storage period. Chokeberry is among the richest plant sources of anthocyanins and possesses one of the highest antioxidant activities (*8*). Sokoł-Łȩtowska *et al.* (*37*) reported that the chokeberry liqueur was the richest in anthocyanins (1674 mg/L) among red fruit liqueurs.

This trend for antioxidant activity over the storage period correlated with the level of antioxidant potential recorded for control (freshly prepared) samples. Mean PC1 scores for the samples after preparation differed significantly (p<0.05) from each other, in the same descending order as observed during the period of storage. Also, regardless of the storage temperature, the curves for chokeberry and apple/chokeberry liqueurs had slightly steeper slopes than apple and apple/beetroot liqueurs, which remained milder (Fig. 2). Although chokeberry and apple/chokeberry liqueurs showed lower total phenolic concentration and antioxidant activity reduction than initial values, according to PC1, the greater initial antioxidant activity expressed through total phenolic concentration, FRAP and DPPH, the greater the rate of their loss during six months of storage at both 4 and 20 °C. Although it has been noticed that sugar moiety stabilises anthocyanins (*46*), they are unstable pigments, easily oxidised, particularly in the presence of ascorbic acid (the most abundant vitamin in the black chokeberry fruit (*8*)) and products of its degradation. Polymerisation and condensation of polyphenols are also believed to be involved in these processes during prolonged storage (*37*). However, due to the variety of compounds interacting simultaneously, it is difficult to establish the exact mechanism of degradation of anthocyanins and other polyphenols.

## CONCLUSIONS

An innovative way of powdered apple, beetroot and chokeberry pomace utilisation was demonstrated. As a source of bioactive molecules, pomace was employed to obtain liqueurs with notable functional and acceptable sensorial properties. According to our knowledge, this is the first study that deals with the application of powdered pomace from industrial juice production in liqueur development. Sensorial properties of freshly produced liqueurs indicated the possibility of chokeberry and apple pomace exploitation in the production of liqueurs without flavour correction, while further research aimed at finding a way to improve sensorial properties of apple with beetroot pomace liqueur and sensory analysis of liqueurs during storage is required. Analysis of individual phenolic compounds revealed the predominance of ellagic acid and phlorizin in freshly prepared liqueurs, except in the chokeberry pomace liqueur in which phlorizin was not quantified. The high total phenolic concentration and antioxidant activity of freshly prepared liqueurs prove that apple, beetroot and chokeberry pomace can be used as a source of bioactive molecules and also indicate the potential contribution of liqueurs to bridging the antioxidant gap in the modern diet. The storability of liqueurs during the initial six months of storage, estimated based on antioxidant activity and total phenolic concentration, showed that they remained a rich source of bioactive compounds despite the significant decrease of surveyed parameters. Measurable changes in colour characteristics were also detected but the appealing colour was retained. Acceptable sensorial properties of freshly prepared liqueurs as well as notable total phenolic concentration and antioxidant activity during 6 months of storage, along with the growing market demand for natural products, indicate the developed products might be an additional source of phytochemicals. The suggested pomace application can also contribute to the adoption of circularity into the fruit and vegetable processing industry.
